# Pulmonary damage caused by lamotrigine

**DOI:** 10.1002/jgf2.322

**Published:** 2020-05-16

**Authors:** Yusuke Mon, Chisato Tamaki

**Affiliations:** ^1^ Department of Internal Medicine Kyoto Kyoritsu Hospital Ayabe City Japan

**Keywords:** drug reaction with eosinophilia and systemic symptoms, drug‐induced hypersensitivity syndrome, lamotrigine, pulmonary damage

## Abstract

This is the first Japanese case of pulmonary damage caused by lamotrigine.

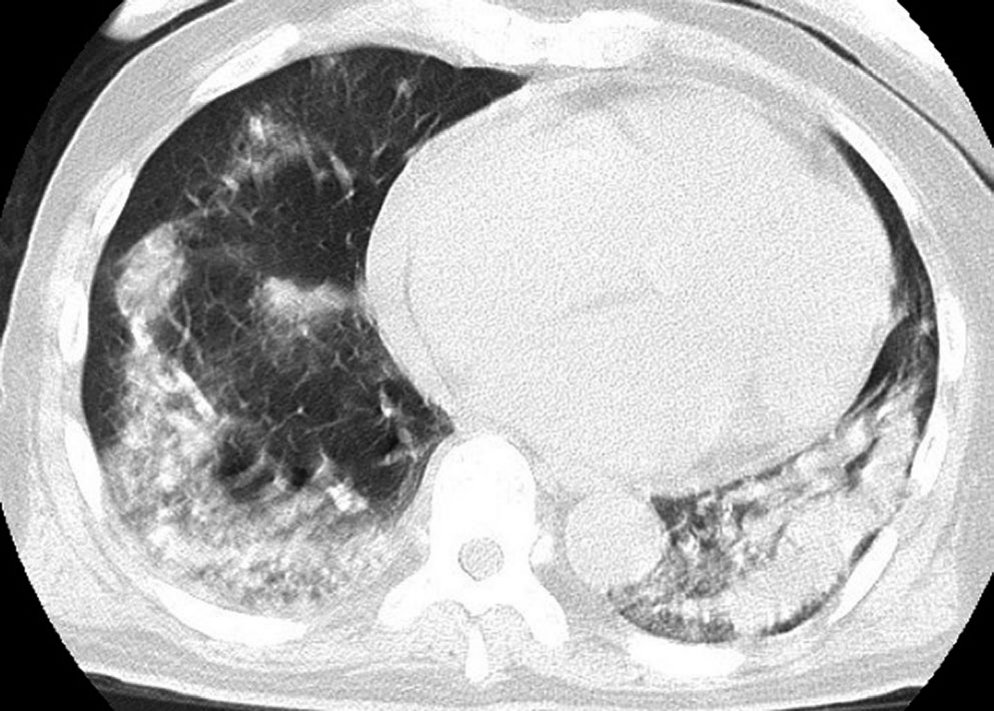

A 69‐year‐old man was admitted to our hospital due to generalized eruption for a week (Figure 1). He had been treated for poststroke epilepsy by lamotrigine, which had been started and increased according to the protocol for 40 days before the advent of eruption.

**Figure 1:**
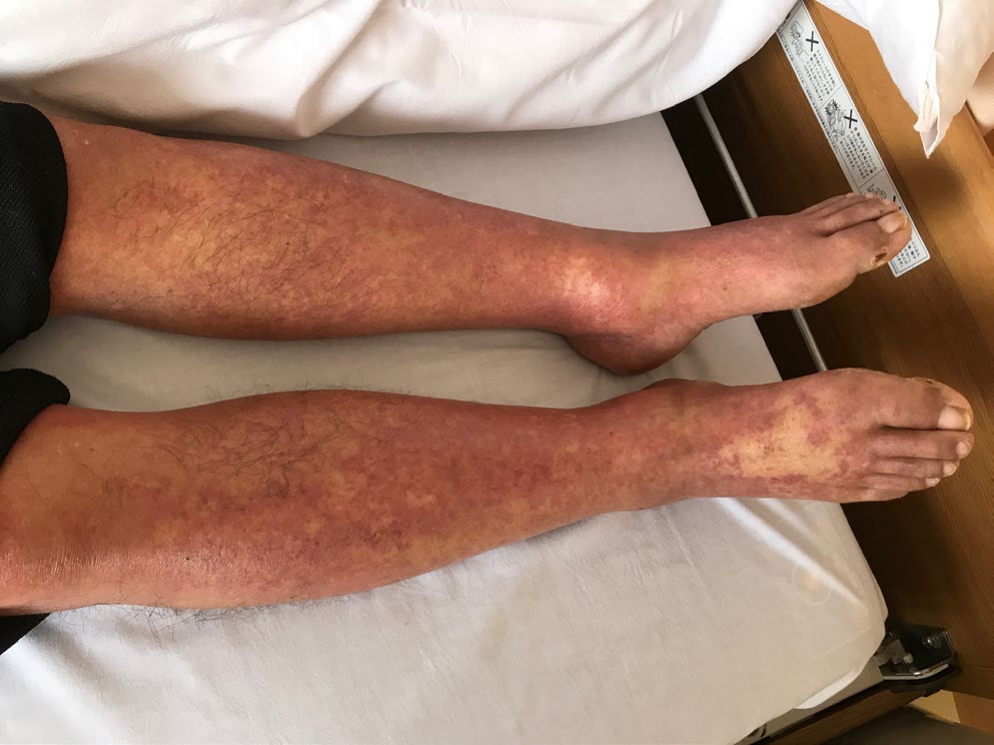
Eruption of lower legs

We stopped lamotrigine and started oral prednisolone 10mg/day. Eruption gradually faded; however, high fever (up to 40°C) appeared, laboratory data showed eosinophilia (1.9 × 10^9^/L, 22.9%) and slight elevations in liver enzymes (AST: 53 U/L, ALT: 31 U/L), and chest CT scan revealed ground glass opacities on both lungs (Figure 2). We started steroid therapy with 1 g of methylprednisolone for 3 days and continued prednisolone for several weeks. The temperature went down, and the ground glass opacities subsided gradually. We did not perform bronchoscopy; therefore, we did not obtain pathological material and bronchoalveolar lavage fluid.

**Figure 2:**
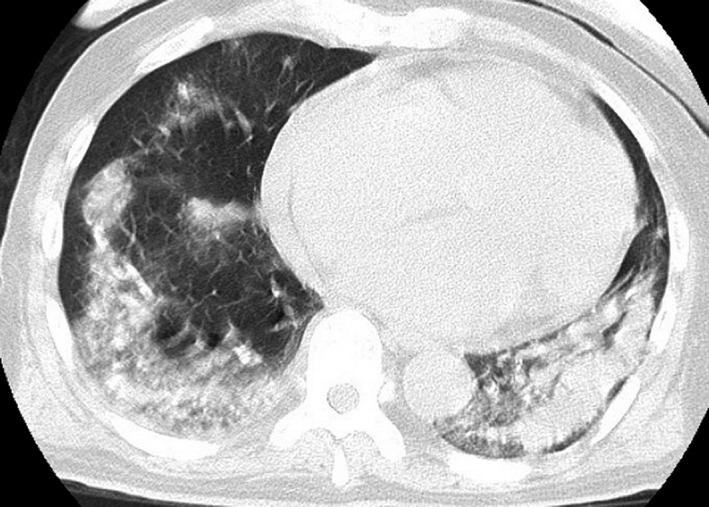
Ground glass opacities on both lungs

Lamotrigine is one of the drugs which cause skin eruption and drug‐induced hypersensitivity syndrome (DiHS)/drug reaction with eosinophilia and systemic symptoms (DRESS)^1^; however, reports of pulmonary damage are rare.^2^ This case satisfied the criteria for DRESS, but not for DiHS. And anti‐HHV‐6 IgG titer (FA) did not significantly change during the interval of two weeks (from 20 to 10). This is the first Japanese case of pulmonary damage caused by lamotrigine.

## CONFLICT OF INTERESTS

The authors have stated explicitly that there are no conflicts of interest in connection with this article.

## PATIENT CONSENT

Patient consent was obtained before publishing this case report.
